# Effect of Microencapsulation on Chemical Composition and Antimicrobial, Antioxidant and Cytotoxic Properties of Lemongrass (*Cymbopogon flexuosus*) Essential Oil

**DOI:** 10.17113/ftb.60.03.22.7470

**Published:** 2022-09

**Authors:** Anely Maciel de Melo, Rafaela Cristina Turola Barbi, Francisco Lucas Chaves Almeida, Weysser Felipe Cândido de Souza, Atacy Maciel de Melo Cavalcante, Hugo Junior Barboza de Souza, Diego Alvarenga Botrel, Soraia Vilela Borges, Roberto Germano Costa, Max Rocha Quirino, Solange de Sousa

**Affiliations:** 1 Department of Chemical Engineering, Federal University of Parana, 81531-990, Curitiba, PR, Brazil; 2 Department of Food Science and Nutrition, School of Food Engineering, University of Campinas; 3 Department of Food Engineering and Technology, School of Food Engineering, University of Campinas; 4 Federal Institute of Education, Science, and Technology of Pernambuco, 55560-000, Barreiros, PE, Brazil; 5 Department Food Science, Federal University of Lavras, 37200-000 Lavras, MG, Brazil; 6 Postgraduate Program in Agro-Food Technology, Federal University of Paraiba, 58225-000, Bananeiras, PB, Brazil

**Keywords:** bioactive properties, gum arabic, maltodextrin, oil retention, spray drying

## Abstract

**Research background:**

Lemongrass (*Cymbopogon flexuosus*) essential oil exhibits antimicrobial and antioxidant properties due to the presence of α-citral and β-citral. Essential oils are susceptible to volatilization and oxidation when applied to food matrices. Therefore, a barrier is needed to protect this material. The present study aims to produce microparticles containing lemongrass essential oil, with gum arabic and maltodextrin using spray drying technology.

**Experimental approach:**

Lemongrass essential oil was extracted by the hydrodistillation method and later microencapsulated with different wall materials. Free and microencapsulated lemongrass essential oil was evaluated for the cytotoxic activity (using *Artemia salina* as test sample), chemical composition (GC-MS), encapsulation efficiency, antioxidant activity (DPPH, ABTS and FRAP), antimicrobial activity and minimum inhibitory concentration.

**Results and conclusions:**

The lethal concentration (LC_50_) of lemongrass essential oil in the cytotoxic test was 8.43 μg/mL against *Artemia salina*; a high activity that can be associated with the presence of α-citral (~33%) and β-citral (~21%) in the samples, since these were the main compounds with bioactive properties. The highest value of microencapsulation efficiency (88.11%) was obtained when only gum arabic was used as wall material. In general, the microparticles showed satisfactory antioxidant activity (expressed as Trolox equivalents, between 348.66 and 2042.30 µmol/100 g) and bactericidal effect *in vitro* against Gram-positive and Gram-negative microorganisms. In conclusion, the microencapsulated lemongrass essential oil is a promising functional additive in the food and pharmaceutical industries.

**Novelty and scientific contribution:**

This study shows that microparticles containing lemongrass essential oil can be prepared using gum arabic and maltodextrin as wall materials by spray drying, resulting in high microencapsulation efficiency. The drying process maintained the antimicrobial and antioxidant properties of the essential oil. Therefore, the microencapsulated lemongrass essential oil is considered a natural, functional and promising additive in the food industry. Its antimicrobial action can increase the shelf life of fresh and semi-fresh products such as cheese, yogurts and meat products. In addition, its antioxidant action can delay the lipid and protein oxidation in food products.

## INTRODUCTION

Lemongrass (*Cymbopogon flexuosus*) is a plant that belongs to the Poaceae family, and it is native to southern Asia and Australia. It is also cultivated in Brazil, commonly found in the states of São Paulo, Minas Gerais, Pará, Pernambuco, Maranhão, Bahia and Rio de Janeiro (*1*). Its essential oil is extracted from the leaves and has been recognized for strong antimicrobial, antifungal and antioxidant capacities (*1*, *2*).

The antimicrobial capacity of lemongrass essential oil is ascribed to the main compounds present in its composition, α-citral and β-citral (*3*, *4*). According to Balti *et al*. (*5*), citral is used in the food industry as food flavoring for beverages, sweets, frozen dairy desserts, baked foods, gelatins, puddings and others. It is also used in cosmetics and perfumery for its lemon scent. However, there are some limitations to the direct application of essential oils rich in bioactive compounds, due to their low stability in food or pharmaceutical products, which is influenced by solvents, pH, temperature, oxygen, light and enzymes (*6*).

Microencapsulation is an alternative process to increase the stability of essential oil, preserving its biological compounds of interest. In general, the technique is based on the formation of emulsion droplets containing the essential oil coated with or incorporated into a homogeneous or heterogeneous matrix, producing small microparticles (*7*).

One of the main benefits of microencapsulation is the increase of the stability of the essential oil, which consists in the formation of a multicomponent structure in the form of microparticles that are composed of a core material and the encapsulant (also called wall material). The main advantage of this process is that it allows sensitive ingredients to be physically trapped in a matrix, therefore, protected against degradation. In the case of essential oils, this mechanism allows the preservation of the compounds present in the oil, responsible for its bioactive properties (*8*).

Spray drying is considered one of the most common methods for obtaining microparticles from a variety of natural raw materials (*9*). The method is relatively easy to execute, it requires few steps to obtain a dry product, and it does not require the use of organic solvents, contributing to a satisfactory cost-benefit ratio. The spray drying has been widely used in food and pharmaceutical processing, aiming to control the release of bioactive compounds (*10*, *11*). Gum arabic and maltodextrin are encapsulating materials generally used in the spray drying, mainly due to their high solubility and low viscosity, which makes them easier to manage (*12*).

In this study, we chose not to use only maltodextrin in the formulations, because despite being a wall material commonly used in industrial spray drying due to its low cost, this material provides low compound retention and reduced emulsifying ability. On the other hand, this material serves as a wall material to microencapsulate essential oils due to its good oxygen barrier properties. However, to improve the physical and chemical properties of the microcapsules, high-molecular-mass materials in the emulsion, such as gum arabic, need to be added. In this context, this work aims to develop and characterize the spray dried microparticles produced with gum arabic and a mixture of gum arabic and maltodextrin as an alternative to improve the stability of the bioactive compounds present in the valuable lemongrass essential oil.

## MATERIALS AND METHODS

### Materials

Gum arabic (Metaquímica Produtos LTDA, Santa Catarina, Brazil) and maltodextrin (MD, DE 9–12; Agro-Industry Commercial Cassava SA, Santa Catarina, Brazil) were used as wall materials to formulate the microparticles. Gallic acid, DPPH˙ (2,2-diphenyl-1-picrylhydrazyl), ABTS (2,2′-azino-bis(3-ethylbenzothiazoline-6-sulfonic acid)), TPTZ (2,4,6-tri(2-pyridyl)-*s*-triazine) and Trolox (6-hydroxy-2,5,7,8-tetramethyl-chroman-2-carboxylic acid) were obtained from Sigma-Aldrich, Merck (St. Louis, MO, USA). The Folin-Ciocalteu reagent was supplied by Merck (Darmstadt, Germany). Fe(III) chloride hexahydrate (FeCl_3_·6H_2_O), sodium acetate trihydrate and sodium carbonate were obtained from Vetec Química Fina Ltda (Duque de Caxias, RJ, Brazil). All reagents used in this work were of analytical grade. Seven bacterial strains, namely *Bacillus cereus* (ATCC 11778), *Campylobacter jejuni* (ATCC 33560), *Clostridium perfringens* (ATCC 3624), *Escherichia coli* (ATCC 10536), *Listeria innocua* (ATCC 33090), *Staphylococcus aureus* (ATCC 23235) and *Salmonella enterica* serotype Typhimurium (ATCC 14028) were used. All microorganisms were from the National Institute for Quality Control and Health (INCQS), associated with the Oswaldo Cruz Foundation (FIOCRUZ), Rio de Janeiro, RJ, Brazil. The brain heart infusion broth and the Müller-Hinton broth were obtained from Merck (Burlington, MA, USA). *Artemia salina* was obtained from the company Artêmia Salina do RN (Natal, RN, Brazil).

### Lemongrass essential oil extraction

The lemongrass leaves were harvested in a rural area located in the city of Serra Redonda, PB, Brazil (S 07°10'42", W 35°40'30"). The essential oil was extracted according to the Brazilian Pharmacopeia (*13*) by steam distillation (model SL76/I; SOLAB, Piracicaba, São Paulo, Brazil). The distillation process was carried out for 3 h, and the temperature was kept at 100 °C. In brief, when the water boiled, the steam passed through the distillation flask, which contained the plant material (14.859 kg). Then, the essential oil was condensed as it passed through a cooled tube. After the distillation, the extracted essential oil was dehydrated with anhydrous granular Na_2_SO_4_, collected in an amber glass vessel and kept under refrigeration at 4 °C until further analysis. The yield was calculated from the oil mass ratio, measured using the density and the volume (mL) of extracted oil, and divided by the dry mass (g) of the sample (*14*), according to the following equation:



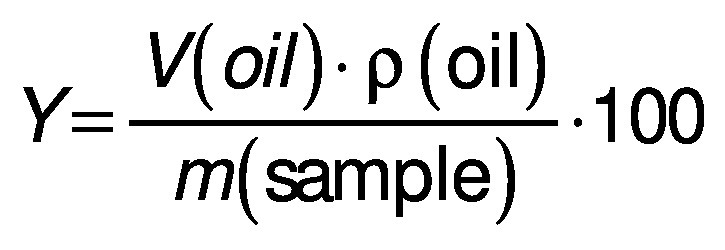



### Cytotoxic activity determined by the lethality test against Artemia salina (brine shrimp)

The toxicity of lemongrass essential oil was determined according to the method described by Meyer *et al*. (*15*). Lemongrass essential oil at different concentrations (from 1 to 200 μg/mL) and solubilized in seawater and Tween 80 was used against *Artemia salina* larvae. First, *Artemia salina* eggs were incubated in seawater under constant aeration at a controlled temperature (25 °C) for 48 h to hatch. Then, ten *Artemia salina* larvae were added to the test tubes containing the lemongrass essential oil solutions and the control solution (without the essential oil). Later, they were incubated in the presence of light for 24 h. After that, the number of surviving larvae in each test tube was counted under a lit background and the lethal concentration (LC_50_) was calculated using the probit method by plotting the concentration of lemongrass essential oil responsible for the death of 50% of the larval population. Data were analyzed in triplicate.

### Microencapsulation of lemongrass essential oil

Two different formulations were used for preparing encapsulant (wall material), one containing only gum arabic and the other containing a blend of gum arabic and maltodextrin (3:1 *m*/*m*), according to the study of de Souza *et al*. (*6*). For each formulation, a solution (20%) of the wall material incorporated with 25% lemongrass essential oil was used. Gum arabic and maltodextrin were dissolved in distilled water under stirring and hydrated for 12 h at 25 °C to ensure complete saturation of the polymer molecules. Then, the lemongrass essential oil was gradually added to the wall material solution and stirred at 3000 rpm for 5 min using a homogenizer (Ultra-Turrax; T18, IKA, Wilmington, NC, USA). The solution was sonicated (Branson Digital Sonifier® model 102C; Branson Ultrasonics Corporation, Danbury, CT, USA) for 2 min at 240 W. The formed emulsion was used as the liquid source in the spray drying. After the drying procedure, the powder was stored in metalized and hermetically sealed packages at 4 °C until further analysis. Data were analyzed in triplicate.

### Chemical composition analysis of free and microencapsulated lemongrass essential oil

The chemical composition of the free and microencapsulated lemongrass essential oil was analyzed by gas chromatography coupled with mass spectrometry (GC-MS), using a gas chromatograph Shimadzu TQ8040 (Shimadzu Corp., Kyoto, Japan), equipped with an RTx-5 capillary column of fused silica (30 m×0.25 mm×0.25 µm) in a similar procedure as described by Lu *et al*. (*16*). First, the oil contained in the microparticles was pre-extracted with *n*-hexane solvent of spectroscopic grade (99.9% purity, Sigma-Aldrich, Merck). For the separation procedure, the initial column temperature was set at 60 °C for 1 min and then increased to 250 °C at a rate of 5 °C/min. Then, the essential oil was diluted in *n*-hexane at a concentration of 0.15 g/L and 5 µL were injected into a GC system *via* split mode injection, with a ratio of 1:30, and an injection temperature of 250 °C and pressure of 57 kPa. Helium was used as a carrier gas at a flow rate of 0.99 mL/min. The mass spectrum was acquired with an ionization energy of 85 eV and a mass scan ranging from 40.0 to 450.0 *m*/*z*. The compounds from the samples were identified based on the library database of the National Institute of Standards and Technology (NIST) (*17*). Data were calculated according to the peak area of ​​the chromatograms and expressed as a percentage.

### Encapsulation efficiency

The encapsulation efficiency (EE) was determined by evaluating the amount of total phenolic compounds in the free and microencapsulated essential oil, according to the method proposed by Cabral *et al*. (*9*) with modifications. The total phenolic compound content was analyzed in the methanolic extracts of the samples using the Folin-Ciocalteu colorimetric method as described by Singleton and Rossi (*18*). The extracts were obtained from 2 mL of the free lemongrass essential oil and 2 g of each microencapsulated lemongrass essential oil, both dissolved in 20 mL of methanol and gently stirred for one hour. After that, the solution was centrifuged at 10 062×*g* for 15 min, and an aliquot of 0.5 mL of the supernatant was collected for analysis. The absorbance was measured at 750 nm in an Epoch microplate spectrophotometer (BioTek, part of Agilent, Winooski, VT, USA) and the total microencapsulated phenolic compounds were expressed in mg of gallic acid equivalents (GAE) per g of sample. Data were analyzed in triplicate. EE was expressed as a percentage of phenolic compounds, according to the following equation:



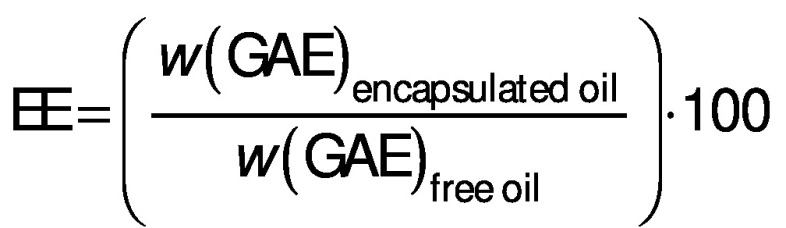



### Microparticle morphology

The microparticles obtained from gum arabic and a mixture of gum arabic and maltodextrin (3:1) were examined by scanning electron microscopy (SEM) using a JEOL JSM 6360-LV microscope (Jeol Company, Tokyo, Japan). The samples were scattered on copper supports using double-sided adhesive tape and then covered with gold coating. The micrographs were obtained under vacuum and at 15 kV of voltage acceleration with magnification under 1200×. The area of the granules was calculated using the ImageJ v. 1.51 software (*19*).

### In vitro antioxidant potential determined by DPPH, FRAP and ABTS assays

The antioxidant potential of the free and microencapsulated lemongrass essential oil was measured in the same methanolic extracts prepared as described in the *Encapsulation efficiency* section, and evaluated using different methods (DPPH, FRAP and ABTS). The DPPH free radical scavenging potential was determined according to the method of Brand-Williams *et al.* (*20*). The analysis was done in a microplate by adding 5 μL of the extract and 195 μL of a 125 μmol/L DPPH methanolic solution. After 30 min of incubation at room temperature in the dark, the absorbance was measured at 517 nm using a spectrophotometer (Synergy; BioTek, part of Agilent) with a microplate reader.

The Fe(III) reducing antioxidant power (FRAP) was determined by the method of Benzie and Strain (*21*). The assay was performed in a microplate by adding 10 μL of the sample and 300 μL of the FRAP reagent in each microwell. After 30 min of incubation at 37 °C in the dark, the absorbance was measured at 593 nm using a spectrophotometer (Synergy; BioTek, part of Agilent) with a microplate reader.

The antioxidant capacity to scavenge ABTS˙^+^ radicals was determined using the method described by Re *et al*. (*22*), with some modifications. The ABTS˙^+^ radical cation was prepared with 7 mmol/L of ABTS and 2.45 mmol/L of potassium persulfate solution (1:1, *V*/*V*). The working solution was stored for 12-16 h at room temperature in the dark. Subsequently, the mixture was diluted with methanol, and the absorbance was adjusted to 0.700±0.020 at 734 nm. Then, 300 µL of the ABTS solution and 3 µL of the sample were added to a microplate. The mixture was stirred and stored in the dark for 30 min. After incubation, the absorbance was measured at 734 nm using a spectrophotometer (Synergy; BioTek, part of Agilent) with a microplate reader. All results were expressed as Trolox equivalents (TE) in µmol of Trolox per 100 g of lemongrass essential oil. The antioxidant analyses were performed using external calibration curves, in which the Trolox ranged from 0.1 to 1.5 µmol/g (FRAP), 0.1 to 1 µmol/g (DPPH) and 0.038 to 2.4 µmol/g (ABTS); the detection limits were (in mmol/g): 1.22 (FRAP), 1 (DPPH) and 2.53 (ABTS).

### Antimicrobial activity determined by disk diffusion agar method

The microorganisms used in this study included bacterial strains selected due to their characteristics as pathogens. The antimicrobial effect of the free and microencapsulated lemon grass essential oil was determined according to the protocol proposed by the Clinical and Laboratory Standards Institute (CLSI) manual (*23*). First, aliquots (100 μL) of each bacterial suspension at 10^8^ CFU/mL previously cultivated in brain heart infusion broth at 37 °C for 24 h were inoculated onto the surface of Petri dishes containing Müller-Hinton agar. Then, three paper discs *d*=6 mm impregnated with 10 μL of free or microencapsulated *φ*(lemongrass essential oil)=1% in distilled water and Tween 80 were evenly distributed on the plates. The plates were incubated at (37±2) °C for 24 h and, after incubation, the diameter of the growth-inhibition zones was measured using a digital caliper. The microorganism resistance to the free and microencapsulated lemongrass essential oil was analyzed according to the size of inhibition halos, which were classified into: resistant (*d*<14 mm), intermediate (*d*=15-19 mm) and susceptible (*d*>20 mm) (*23*).

### Determination of minimum inhibitory concentration

The minimum inhibitory concentration (MIC) assay was performed according to the method described in CLSI (*23*). The bacterial suspensions (10^8^ CFU/mL) were inoculated onto the surface of Petri dishes containing Müller-Hinton agar. Then, disks impregnated with the free and microencapsulated lemongrass essential oil at volume fractions ranging from 1 to 6% and a control (without the essential oil) were placed on the plates, which were incubated at 37 °C for 24 h. After incubation, the disk with the lowest volume fraction of the essential oil that inhibited the visible bacterial growth was considered the MIC value. The MIC value was analyzed in triplicate.

### Statistical analysis

The data of three replicates were statistically investigated by one-way analysis of variance (ANOVA) using the SAS® v. 9.0 software (*24*) licensed to the Federal University of Paraíba, Brazil, and the significant differences between treatments were analyzed using the Sheffé’s test when p-values were lower than 0.05. Pearson’s correlation coefficient was used to determine the correlation between the antioxidant methods and total phenolic compounds.

## RESULTS AND DISCUSSION

### Extraction yield of lemongrass essential oil

The yield of lemongrass essential oil was 0.45% (data not shown), corresponding to 67 mL of oil extracted by steam distillation, which is lower than the yields reported in the literature obtained by hydrodistillation, which varied between 0.70 and 0.95% (*4*, *5*). This smaller yield obtained in this study may be related to the lemongrass variety, climate characteristics of the region where the lemongrass was produced, stage of plant development and extraction conditions (*5*), factors that alter plant metabolism. It is important to emphasize that although the extracted lemongrass essential oil has a lower yield than previous findings in the literature, its antioxidant and antimicrobial properties may justify future industrial applications.

### Cytotoxic activity of essential oil determined with Artemia salina

The *Artemia salina* toxicity test is a biological test considered one of the tools used for the preliminary toxicity assessment of bioactive compounds (*25*). According to Meyer *et al*. (*15*), this test is an efficient bioassay that can be widely used by pharmacologists and chemists to detect and isolate plant constituents with biological properties. Moreover, it is practical, fast, safe, economical and has a good correlation with cytotoxic activity in some tumors, as well as anticancer, insecticide, molluscicide and antifungal activities (*26*, *27*). In this sense, compounds considered toxic for *Artemia* cells may show some other biological properties, such as antioxidant and antimicrobial.

The values of lethal concentration (LC) of lemongrass essential oil obtained in this study were (in μg/mL): LC_50_=5.31−13.97, LC_90_=14.29−90.25 and LC_95_=17.81−162.75 (data not shown). There was a 100% lethality of microcrustaceans when 200 μg/mL of essential oil was used. The toxicity is considered low when LC_50_≥500 μg/mL, intermediate when 100≤LC_50_≤500 μg/mL, and toxic when LC_50_≤100 μg/mL (*28*). The LC_50_ toxicity of lemongrass essential oil can be attributed to the major compounds, mainly to the content of citral isomers, since they show great biological properties including cytotoxicity, as already reported in other studies (*29*-*31*). Although the use of this essential oil in the food industry can benefit consumers, the applied amount should be carefully considered.

### Morphology of the microparticles evaluated by scanning electron microscopy

The efficiency of a microencapsulation process can be indirectly evaluated by analyzing the morphology of the microparticles since structures with cracks or damages can compromise the carrying and the protection of the microencapsulated compound of interest (*8*).

**Fig. 1:**
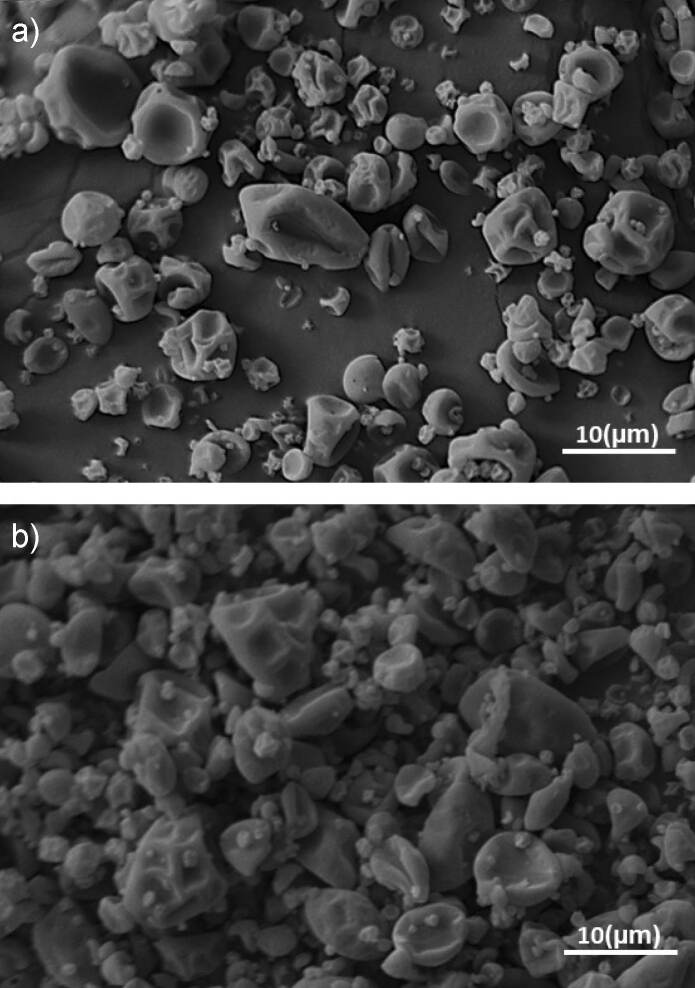
Micrographs of microencapsulated lemon grass essential oil: a) gum arabic and b) a mixture of gum arabic and maltodextrin

Fig. 1 shows the microparticles prepared with pure gum arabic and a mixture of *m*(gum Arabic):*m*(maltodextrin)=3:1. Images of the microparticles show that there was no evidence of cracks on the particle surfaces, ensuring low gas permeability and better protection of the lemongrass essential oil. This is an important feature for microencapsulated essential oil as it ensures that their volatile compounds remain protected for a longer period, being released in a controlled manner when introduced into the food matrix.

A wrinkled structure was observed in both treatments, and it represents a characteristic of microparticles stabilized by the spray drying (*6*). Therefore, differences in the wall material formulation did not affect the morphological characteristics of the microparticles. As is observable in Fig. 1a, the microparticles produced using only gum arabic have more smooth granules than those produced using maltodextrin (Fig. 1b).

### Chemical composition determined by GC-MS

Different compounds, *e.g*. ketones, fatty aldehydes, alkenes and terpenoids, were identified by analyzing the chemical composition of the free and microencapsulated essential oil. The component profiles (%) are shown in Table 1.

**Table 1:** Major compounds of lemongrass (*Cymbopogon flexuosus*) essential oil and its microcapsules

Compound	Lemongrass essential oil			Oil microcapsules with gum arabic			Oil microcapsules with *m*(gum arabic)/*m*(maltodextrin)=3:1	
Area/%	*t*_r_/min		Area/%	*t*_r_/min		Area/%	*t*_r_/min
2-Hexanone	1.11	3.06		1.11	3.06		1.61	3.05
3-Methylcyclopentanone	0.67	3.96		0.40	3.95		0.63	3.94
2,4-Hexadienal	0.06	4.53		0.04	4.51		0.07	4.51
1-Hexyn-3-ol	0.52	5.00		0.52	4.98		0.61	4.97
2,4-Dimethyl-2-pentene	3.96	5.13		1.35	5.20		1.62	5.19
2-Nitrohexane	5.78	5.57		1.87	6.11		2.34	6.10
α-Pinene	0.11	5.87		n.d.	n.d.		0.08	5.84
Camphene	0.68	6.30		0.47	6.28		0.65	6.27
3-Butenyl hexyl ether	1.44	6.40		n.d.	n.d.		n.d.	n.d.
3-Hexen-2-one	4.67	6.80		5.48	6.77		5.95	6.76
Myrcene	0.28	7.45		0.18	7.44		0.24	7.42
6-Methyl-5-hepten-2-one	1.07	7.32		0.69	7.30		0.78	7.29
l-Limonene	0.93	8.79		0.59	8.76		0.74	8.75
Linalool	1.55	11.47		1.59	11.44		1.50	11.43
Citronellal	0.06	13.72		0.03	13.68		0.05	13.69
Isogeranial	0.35	14.23		0.29	14.19		0.31	14.19
β-Citral	22.92	17.64		22.41	17.60		21.80	17.58
α-Citral	36.70	18.98		36.28	18.95		33.12	18.92
Caryophyllene	2.57	25.54		0.31	25.50		0.38	25.49
α-Humulene	0.19	27.00		n.d.	n.d.		n.d.	n.d.
γ-Cadinene	0.48	29.92		n.d.	n.d.		n.d.	n.d.
Caryophyllene oxide	0.51	32.38		0.39	32.33		0.23	32.33
Other compouds	12.85	-		25.30	-		27.26	-
Total	99.46			99.29			99.97	

*t*_r_=retention time, n.d.=not detected

The main compounds identified in the lemongrass essential oil and in the microparticles with gum arabic or mixture of gum arabic and maltodextrin were α-citral (36.70, 36.28 and 33.12% respectively), followed by β-citral (22.92, 22.41 and 21.80% respectively). Previous studies stated that the citral is the main compound responsible for the antimicrobial activity of lemongrass essential oil (*3*, *32*). Some components in low amounts were detected only in the non-microencapsulated essential oil, such as 3-butenyl hexyl ether, α-humulene and γ-cadinene. These compounds may have been evaporated during the heat treatment.

It is observable from the obtained results that the main components of interest (α- and β-citral) were retained after the drying, with a significant percentage of the oil components. Although some amount of essential oil may be lost due to the high temperatures during the spray drying, some of the main components of the oil are still present in the microparticles containing the essential oil. Therefore, future industrial applications of lemongrass essential oil using gum arabic or the mixture of gum arabic and maltodextrin can be considered viable, since these encapsulation materials have shown to be efficient in retaining most of the compounds identified in the non-microencapsulated essential oil.

### Encapsulation efficiency of lemongrass essential oil

The encapsulation efficiency (EE) of lemongrass essential oil significantly differed (p<0.05) between the encapsulating materials (Table 2). The gum arabic microparticle had an efficiency of about 88% in the microencapsulation of lemongrass essential oil, while the mixture of gum arabic and maltodextrin had 71% efficiency. Similar results were found in the study of Rajabi *et al*. (*33*), where the same trend was observed during encapsulation of the bioactive components of saffron by spray drying using gum arabic, maltodextrin and gelatin as wall materials. Garcia *et al*. (*34*) also found a similar efficiency level (73.57%) during the microencapsulation of basil essential oil using a gum arabic-based formulation. In general, the spray drying used to encapsulate essential oil can maintain the volatile components during drying (*6*).

**Table 2:** Encapsulation efficiency and antioxidant activity of free and microencapsulated lemongrass essential

Sample		*b*(TE)/(µmol/100 g)			
EE/%	ABTS	DPPH	FRAP
Lemongrass essential oil	-		(2353±3)^a^	(929.1±0.2)^a^	(1108.6±0.2)^a^
Oil microcapsules with gum arabic	(88.1±3.0)^a^		(2042.30±0.05)^b^	(868±4)^b^	(906.3±0.3)^b^
Oil microcapsules with *m*(gum arabic)/*m*(maltodextrin)=3:1	(71.1±0.2)^b^		(1108.6±0.3)^c^	(348.7±1.2)^c^	(630.4±1.0)^c^


Data were analyzed in triplicate and expressed as mean value±S.D. Mean values followed by the same letter in the same column do not differ significantly (p>0.05) according to Sheffè test. EE=encapsulation efficiency. TE=Trolox equivalents oil

It was observed in this study that the addition of maltodextrin in the microparticle formulation led to a decrease in the encapsulation efficiency. This result may be related to the interaction between the bioactive compounds present in the essential oil and the wall materials used as encapsulants (*8*). Likewise, the matrix formed using gum arabic and maltodextrin was not compact enough to retain a higher amount of lemongrass essential oil than with the gum arabic matrix. Furthermore, the temperature of the drying air used in the spray dryer can also influence the encapsulation efficiency (*8*).

### Antioxidant capacity of free and microencapsulated lemongrass essential oil

The results of the antioxidant activity of lemongrass essential oil and the microparticles that contain it are shown in Table 2. As expected, our results for antioxidant activity in ABTS, DPPH and FRAP assays were higher for the non-microencapsulated lemongrass essential oil. Although the microparticles had shown lower antioxidant capacity than the free oil, the microencapsulation was able to maintain the bioactivity of the compounds. Consequently, the microencapsulation technology can keep the compounds stable during storage, maintaining their antioxidant property (*11*, *35*).

As described in Table 2, there was a significant difference (p<0.05) between the used encapsulating materials. The results for gum arabic microparticles were better than for the microparticles of the gum arabic and maltodextrin mixture. It was observed that lemongrass essential oil microencapsulated in gum arabic had an antioxidant capacity, expressed as TE, of 2042.30, 868 and 906.3 µmol/100 g in ABTS, DPPH and FRAP assays, respectively, compared to the microencapsulated oil in the mixture of gum arabic and maltodextrin.

Comparing the lemongrass essential oil with the essential oil from other species (*Cymbopogon citratus*), Fokom *et al*. (*2*) obtained lower values of the antioxidant activity determined with DPPH assay. These authors state that the antioxidant activity of the essential oil varies with the harvest period and symbiotic status of the plant used to extract the essential oil, which can also cause variation among different species. The objective of microencapsulating the lemongrass essential oil is to reduce these variations, due to the protection caused by the wall material that retains compounds when compared to the free essential oil. Da Silva *et al*. (*36*) showed that gum arabic, as a wall material, showed better action in preserving bioactive compounds in spray dried products than other carriers. The same behavior was exhibited in this study. This is possibly due to the affinity of gum arabic for the bioactive compounds present in the samples under study, leading to a thermoprotective effect during exposure to higher temperatures.

### *Antimicrobial activity* of *free and microencapsulated lemongrass essential oil*

The antimicrobial activities of the free and microencapsulated lemongrass essential oil are shown in Table 3. According to the classification described by the CLSI (*23*), the strains of *C. perfringens*, *S.* Typhimurium and *L. innocua* were resistant to the lemongrass essential oil microencapsulated in gum arabic, while the Gram-negative bacteria *E. coli* and *S.* Typhimurium were resistant to the oil microencapsulated in the mixture of gum arabic and maltodextrin.

**Table 3:** Antibacterial activity of lemongrass essential oil and its microcapsules

Microorganism	Treatment										
Lemongrass essential oil				Gum arabic					*m*(gum arabic):*m*(maltodextrin)=3:1	
*d*(inhibition)/mm	MIC/ (µg/mL)		*d*(inhibition)/mm		MIC/ (µg/mL)		*d*(inhibition)/mm			MIC/ (µg/mL)
Gram-positive											
*B. cereus*	(27.3±0.6)^a^	10.10		(14.5±1.5)^b^		10.10		(12.0 ±1.0)^b^			10.10
*C. perfringens*	(19.5±1.5)^a^	20.41		(13.5±0.5)^b^		30.92		n.d.			n.d.
*L. innocua*	(14.1±0.8)^a^	30.92		(12.5±0.5)^b^		10.10		n.d.			n.d.
*S. aureus*	(19.3 ±1.5)^a^	10.10		(14.8±0.8)^b^		20.41		(12.3±0.6)^b^			30.92
Gram-negative											
*C. jejuni*	(22.8±0.3)^a^	10.10		(18.5 ±0.5)^c^		20.41		(20.0±1.4)^b^			20.41
*E. coli*	(18.0±1.0)^a^	20.41		(14.0±2.0)^b^		30.92		(12.77±1.2)^b^			30.92
*S.* Typhimurium	(14.7±0.8)^a^	30.92		(13.6± 2.1)^ab^		41.67		(10.5±0.5)^c^			63.83


Data were analyzed in triplicate and expressed as mean value±S.D. Mean values followed by the same letter in the same row do not differ significantly (p>0.05) according to Sheffè test. MIC=minimal inhibitory concentration, n.d.=not detected

*B. cereus* and *C. jejuni* were the most sensitive strains to the free lemongrass essential oil, presenting inhibition zones greater than 20 mm, and therefore, were classified as susceptible (*25*). The citral contents can be associated with the high efficacy of the oil against these microorganisms. Yoplac *et al*. (*37*) studied the antimicrobial activity of citral and observed that the lowest concentration used (0.80 mg/mL) inhibited *B. cereus*. In another study, strains of *C. jejuni* were inhibited by a low amount of lemongrass essential oil (0.018%), and this activity was attributed to the citral content (*38*). Both authors reported that citral can alter and penetrate the bacterial cell wall, leading to protein denaturation and cell membrane destruction, thus causing lysis and cell death.

The inhibitory effect of the lemongrass essential oil was reduced after microencapsulation, which can be associated with the wall material of microcapsules. This material controls the release of the essential oil and its bioactive compounds due to the barrier the material creates. Thus, probably the exposure time of microorganisms to the microencapsulated essential oil was not sufficient for their inhibition.

The strains of *L. innocua* and *C. perfringens* were not inhibited by the lemongrass essential oil microencapsulated in the mixture of gum arabic an maltodextrin, so an inhibition zone was not formed. De Souza *et al.* (*6*) studied the antimicrobial effect of orange essential oil microencapsulated in gum arabic and maltodextrin. They reported that the addition of maltodextrin reduced the solubility of the microparticles. This lower solubility probably caused low antimicrobial activity because the mechanism of action of lemongrass essential oil on these microorganisms is through their membrane permeabilization (*39*). Thus, the passage of the essential oil to the cell periplasm of the microorganisms was prevented. In addition, the microparticles of the mixture of gum arabic and maltodextrin showed lower encapsulation efficiency (Table 2).

Studies evaluating the antimicrobial activity of microcapsules of lemongrass essential oil prepared with gum arabic and maltodextrin as encapsulating materials have not been conducted. Leimann *et al.* (*3*) analyzed the essential oil of lemongrass (*Cymbopogon citratus*) and confirmed that citral is its main compound. In that study, which used poly(vinyl alcohol) crosslinked in glutaraldehyde under acidic conditions as an encapsulating material, they found that free and microencapsulated essential oil showed the same MIC for *E. coli* (22.32 mg/mL) and *S. aureus* (2.79 mg/mL), meaning that the encapsulation process did not cause deterioration of the essential oil.

The values of the MIC test confirmed the results obtained with the agar diffusion method. Lemongrass essential oil microencapsulated in the mixture of gum arabic and maltodextrin did not show antimicrobial activity against the strains of *C. perfringens* and *L. innocua* (Table 3). On the other hand, *B. cereus* and *C. jejuni* were inhibited at the lowest tested free lemongrass essential oil concentration (10.10 µg/mL). In addition, both microcapsules containing the lowest concentration of essential oil inhibited *B. cereus*. These results confirm the high sensibility of the *B. cereus* and *C. jejuni* strains to the lemongrass essential oil, as already reported in other studies (*37*, *38*).

Due to the greater inhibition zone for *E. coli*, the free lemongrass essential oil was more efficient than the microencapsulated oil. Both microencapsulated oils had a MIC value of 30.92 µg/mL. Even though the microencapsulated lemongrass essential oil samples were less effective than the free oil, our results were better than those reported by Assis *et al*. (*40*), who studied the antimicrobial activity of the free and microencapsulated lemongrass essential oil against *E. coli*. The authors obtained a MIC of 80 µL/mL. In another study, the antimicrobial activity of lemongrass essential oil and citral was evaluated against *E. coli* (*41*). The authors reported high inhibition of the microorganism at low concentrations with MIC values of 2.2 and 1.0 mg/mL of the oil and citral, respectively. They also reported that the high activity is related to the mechanism of action of the essential oil and citral, which can degrade membrane proteins and increase the cell permeability of microorganisms.

The free lemongrass essential oil was more effective against *S. aureus*, with a MIC value of 10.10 µg/mL, than the microcapsules containing the oil, which had MIC values between 20.41 and 30.92 µg/mL. The differences between the MIC values were influenced by the microencapsulation process as well as the used wall materials. Overall, the microparticles produced with gum arabic had a better effect than the microparticles made using the mixture of gum arabic and maltodextrin (3:1) as wall materials.

## CONCLUSIONS

The results obtained in this study show that microparticles containing lemongrass essential oil can be prepared by spray drying using gum arabic and maltodextrin as wall materials, resulting in high microencapsulation efficiency, with gum arabic being more effective in oil retention. In addition, the essential oil was not degraded by spray drying, demonstrating the efficacy of the gum to encapsulate the oil and retain its compounds. The microparticles prepared using gum arabic had a more regular surface, higher antioxidant property and better retention of citral compounds. The microparticles maintained antibacterial and antioxidant properties when compared to the free essential oil, which is of great interest to the food and pharmaceutical industries. In general, gum arabic is considered a good encapsulating material for lemongrass essential oil for future elaboration of microparticles with industrial applications.
